# Supplementary material for: The Prevalence of Onchocerciasis-Associated Epilepsy in Mundri West and East Counties, South Sudan: A Door-to-Door Survey
Source: Pathogens. 2022 Mar 24;11(4):396. doi: 10.3390/pathogens11040396 (PMC9025150; doi:10.3390/pathogens11040396)
Supplement: Supplementary file 1 [file pathogens-11-00396-s001.zip › pathogens-1628006-supplementary.pdf]

HOUSEHOLD SURVEY QUESTIONNAIRE: SCREENING FOR EPILEPSY

Investigator's Name: ____ Signature:______________________

Village: ______________________________ Date current visit (DD/MM/YYYY): __ __/__ __/__ __ __ __

Previous HOUSEHOLD CODE __ __ __/ __ __/__ __ __ Date previous visit __ __/__ __/__ __ __ __ GPS____________________________

1) Name of Household head **(*)**: ____ Tel: ________ Etnicity__________________

2) Does family originate from this village?: ☐ YES ☐ NO If NO, How long have they been residing in this village? ____________ (years)

3) Main income generating activity of the family: several answers possible

Farming cattle pigs fishing shop employe other, specify______________________

4) Has anybody died in the household since last survey?

If YES, Name : ____age ___________sex______When _____month_____year Cause? Epilepsy/nodding Other

If YES, Name : ____age ___________sex______When _____month_____year Cause? Epilepsy/nodding Other

If YES, Name : ____age ___________sex______When _____month_____year Cause? Epilepsy/nodding Other

If YES, Name : ____age ___________sex______When _____month_____year Cause? Epilepsy/nodding Other

4) Has anybody developed epilepsy/nodding in the household since last survey?

If YES, Name : ____age ___________sex______When _____month_____year

If YES, Name : ____age ___________sex______When _____month_____year

If YES, Name : ____age ___________sex______When _____month_____year

If YES, Name : ____age ___________sex______When _____month_____year

(*) Household definition: *All occupants of the same home with or without parental ties who take meals together*

EPILEPSY SCREENING: If at least one response for 4.1 - 4.5 is YES, refer the participant to a neurologist. [YES: Yes; NO: No; ? : Unknown

1. No. pers.		2. Age	3. Gender		4.1 QUESTION 1			4.2 QUESTION 2			4.3 QUESTION 3			4.4 QUESTION 4			4.5 QUESTION 5		
Pst	Abs	Year: Y<br>Month: M	M=Male<br>F=Female		Loss of consciousness with either urine on self and/or drooling?			Absence(s) or loss of contact with the surrounding of sudden onset and brief duration?			Jerking or uncontrolled abnormal movement (convulsion) of the limb(s) of sudden onset and lasting for a few minutes?			Head nodding?			Has it ever been said that he/she is epileptic or has he already had 2 or more seizures?		
1 ☐	☐		M ☐	F ☐	YES ☐	NO ☐	? ☐	YES ☐	NO ☐	? ☐	YES ☐	NO ☐	? ☐	YES ☐	NO ☐	? ☐	YES ☐	NO ☐	? ☐
2 ☐	☐		M ☐	F ☐	YES ☐	NO ☐	? ☐	YES ☐	NO ☐	? ☐	YES ☐	NO ☐	? ☐	YES ☐	NO ☐	? ☐	YES ☐	NO ☐	? ☐
3 ☐	☐		M ☐	F ☐	YES ☐	NO ☐	? ☐	YES ☐	NO ☐	? ☐	YES ☐	NO ☐	? ☐	YES ☐	NO ☐	? ☐	YES ☐	NO ☐	? ☐
4 ☐	☐		M ☐	F ☐	YES ☐	NO ☐	? ☐	YES ☐	NO ☐	? ☐	YES ☐	NO ☐	? ☐	YES ☐	NO ☐	? ☐	YES ☐	NO ☐	? ☐
5 ☐	☐		M ☐	F ☐	YES ☐	NO ☐	? ☐	YES ☐	NO ☐	? ☐	YES ☐	NO ☐	? ☐	YES ☐	NO ☐	? ☐	YES ☐	NO ☐	? ☐
6 ☐	☐		M ☐	F ☐	YES ☐	NO ☐	? ☐	YES ☐	NO ☐	? ☐	YES ☐	NO ☐	? ☐	YES ☐	NO ☐	? ☐	YES ☐	NO ☐	? ☐
7 ☐	☐		M ☐	F ☐	YES ☐	NO ☐	? ☐	YES ☐	NO ☐	? ☐	YES ☐	NO ☐	? ☐	YES ☐	NO ☐	? ☐	YES ☐	NO ☐	? ☐
8 ☐	☐		M ☐	F ☐	YES ☐	NO ☐	? ☐	YES ☐	NO ☐	? ☐	YES ☐	NO ☐	? ☐	YES ☐	NO ☐	? ☐	YES ☐	NO ☐	? ☐
9 ☐	☐		M ☐	F ☐	YES ☐	NO ☐	? ☐	YES ☐	NO ☐	? ☐	YES ☐	NO ☐	? ☐	YES ☐	NO ☐	? ☐	YES ☐	NO ☐	? ☐
10 ☐	☐		M ☐	F ☐	YES ☐	NO ☐	? ☐	YES ☐	NO ☐	? ☐	YES ☐	NO ☐	? ☐	YES ☐	NO ☐	? ☐	YES ☐	NO ☐	? ☐
11 ☐	☐		M ☐	F ☐	YES ☐	NO ☐	? ☐	YES ☐	NO ☐	? ☐	YES ☐	NO ☐	? ☐	YES ☐	NO ☐	? ☐	YES ☐	NO ☐	? ☐
12 ☐	☐		M ☐	F ☐	YES ☐	NO ☐	? ☐	YES ☐	NO ☐	? ☐	YES ☐	NO ☐	? ☐	YES ☐	NO ☐	? ☐	YES ☐	NO ☐	? ☐
13 ☐	☐		M ☐	F ☐	YES ☐	NO ☐	? ☐	YES ☐	NO ☐	? ☐	YES ☐	NO ☐	? ☐	YES ☐	NO ☐	? ☐	YES ☐	NO ☐	? ☐
14 ☐	☐		M ☐	F ☐	YES ☐	NO ☐	? ☐	YES ☐	NO ☐	? ☐	YES ☐	NO ☐	? ☐	YES ☐	NO ☐	? ☐	YES ☐	NO ☐	? ☐
15 ☐	☐		M ☐	F ☐	YES ☐	NO ☐	? ☐	YES ☐	NO ☐	? ☐	YES ☐	NO ☐	? ☐	YES ☐	NO ☐	? ☐	YES ☐	NO ☐	? ☐
16 ☐	☐		M ☐	F ☐	YES ☐	NO ☐	? ☐	YES ☐	NO ☐	? ☐	YES ☐	NO ☐	? ☐	YES ☐	NO ☐	? ☐	YES ☐	NO ☐	? ☐
17 ☐	☐		M ☐	F ☐	YES ☐	NO ☐	? ☐	YES ☐	NO ☐	? ☐	YES ☐	NO ☐	? ☐	YES ☐	NO ☐	? ☐	YES ☐	NO ☐	? ☐
18 ☐	☐		M ☐	F ☐	YES ☐	NO ☐	? ☐	YES ☐	NO ☐	? ☐	YES ☐	NO ☐	? ☐	YES ☐	NO ☐	? ☐	YES ☐	NO ☐	? ☐
19 ☐	☐		M ☐	F ☐	YES ☐	NO ☐	? ☐	YES ☐	NO ☐	? ☐	YES ☐	NO ☐	? ☐	YES ☐	NO ☐	? ☐	YES ☐	NO ☐	? ☐
20 ☐	☐		M ☐	F ☐	YES ☐	NO ☐	? ☐	YES ☐	NO ☐	? ☐	YES ☐	NO ☐	? ☐	YES ☐	NO ☐	? ☐	YES ☐	NO ☐	? ☐

PERSONS SUSPECTED TO HAVE EPILEPSY (REFER TO DOCTOR / NEUROLOGIST) :

CODE (I/VV/HHH/N°): __ __ __/__ __/__ __ __/__ _ Name: ___________________________________ Sex (M/F): ____ Age: ________

CODE (I/VV/HHH/N°): __ __ __/__ __/__ __ __/__ _ Name: ___________________________________ Sex (M/F): ____ Age: ________

CODE (I/VV/HHH/N°): __ __ __/__ __/__ __ __/__ _ Name: ___________________________________ Sex (M/F): ____ Age: ________

CODE (I/VV/HHH/N°): __ __ __/__ __/__ __ __/__ _ Name: ___________________________________ Sex (M/F): ____ Age: ________
